# Hepatic Steatosis Alleviated by a Novel Metformin and Quercetin Combination Activating Autophagy Through the cAMP/AMPK/SIRT1 Pathway

**DOI:** 10.5812/ijpr-136952

**Published:** 2023-07-10

**Authors:** Havva Afshari, Shokoofe Noori, Afshin Zarghi

**Affiliations:** 1 Department of Clinical Biochemistry, Faculty of Medicine, Shahid Beheshti University of Medical Sciences, Tehran, Iran; 2 Department of Pharmaceutical Chemistry, School of Pharmacy, Shahid Beheshti University of Medical Sciences, Tehran, Iran

**Keywords:** Hepatic Steatosis, Metformin, Quercetin, Autophagy, SIRT1, cAMP, AMPK

## Abstract

Non-alcoholic fatty liver disease (NAFLD) incidence and prevalence are rapidly increasing globally. The combined effects of metformin and quercetin (Que) have yet to be investigated. However, both have demonstrated the potential to reduce triglyceride (TG) levels and treat NAFLD by promoting autophagy. The objective of the present study was to elucidate the mechanism of action and assess the role of autophagy in the lipid-lowering effects of Que, both individually and in combination with metformin, in a HepG2 cell model of hepatic steatosis. Triglyceride levels and lipogenic gene expression were reduced in HepG2 cells exposed to palmitic acid (PA) when treated with Que-metformin, as evidenced by triglyceride measurements and real-time PCR. The LDH release assay also showed that this combination induced autophagy to protect HepG2 cells from PA-induced cell death. According to the Western blot analysis outcomes, Que-metformin increased LC3-I and LC3-II protein levels while decreasing p62 expression to induce autophagy. In HepG2 cells, the co-administration of Que-metformin elevated cAMP, phosphorylated AMP-activated protein kinase (p-AMPK), and Beclin-1 levels. Additionally, the inhibition of SIRT1 reversed the autophagy induced by Que-metformin. The findings of this study demonstrated for the first time that Que-metformin reduced hepatosteatosis by stimulating autophagy through the cAMP/AMPK/SIRT1 signaling pathway and diminishing inflammatory cytokines.

## 1. Background

Non-alcoholic fatty liver disease (NAFLD) is characterized by an elevated accumulation of fats within the liver. It is associated with serious liver diseases such as non-alcoholic steatohepatitis (NASH), cirrhosis, hepatic steatosis, and hepatocellular carcinoma (HCC), in addition to other metabolic illnesses like diabetes (1). The prevalence of NAFLD is quickly increasing worldwide, and its etiology is complicated and poorly understood. However, it includes changes in energy metabolism, insulin resistance, inflammatory processes, and intrahepatic lipid accumulation (IHL) (2). Therefore, there is an urgent need to gain knowledge of NAFLD molecular mechanisms and discover new drugs for treatment.

The most prevalent antioxidants in the human diet are known as polyphenols, which have long been associated with preventing and treating several ailments. Studies have revealed that polyphenols can control de novo lipogenesis by influencing lipogenic enzyme activity and enhancing lipolytic protein expression (3). Quercetin (Que) is a plentiful polyphenolic flavonoid (3,3,4’,5,7-pentahydroxyflavone), possessing anti-inflammation, anti-apoptosis, immunoprotection, and anti-cancer properties. Evidence from both in vivo and in vitro studies shows that quercetin at a moderate dose protects the liver at various stages of NAFLD by limiting intrahepatic fat buildup, lowering inflammatory factors, and delaying the fibrotic process. As a natural phytochemical shown to have minimal toxicity and few adverse effects, Que is a promising candidate for use as an additional additive to treat NAFLD (4).

Metformin is the primary therapy recommended as a first-line approach for type 2 diabetes, specifically in individuals who are overweight or have obesity (5). Due to its reduced hepatocyte lipid production and insulin-sensitizing actions, it is also suggested as a viable treatment for NAFLD (6, 7). However, for the best outcomes, substantial dosages of metformin are frequently required (8). This medication also has unfavorable adverse effects, such as diarrhea, liver toxicity, and lactic acidosis (9). Studies have shown that combining metformin with other drugs, as opposed to monotherapy, can enhance metformin’s effectiveness for treating NAFLD in animal models (10).

Autophagy is a cellular mechanism that facilitates the breakdown of damaged or surplus cellular components, including lipids. Autophagy has been reported to regulate the breakdown of intracellular lipids in hepatocytes and, consequently, can control the emergence of hepatic steatosis and the pathophysiology of NAFLD. In the case of NAFLD, hepatocytes tend to accumulate excessive amounts of triglycerides and cholesterol in lipid droplets, and autophagy can modulate this hepatocellular lipid accumulation by selectively breaking down these lipids. Through this process, autophagy can impede the progression of NAFLD by promoting lipid breakdown and reducing the extent of hepatic steatosis (11). Therefore, targeting autophagy may provide a new avenue for developing effective treatments for NAFLD. Sirtuin 1 (SIRT1) is the primary autophagy regulator and senses metabolic stress and nutritional status (12). SIRT1/AMP-activated protein kinase (AMPK) controls one another and collaborates on downstream signaling molecules that control various biological functions, including cellular energy metabolism, the inflammatory response, and mitochondrial function (13). The activation of SIRT1/AMPK is a cyclic amplification mechanism. Reduced cellular energy promotes AMPK, which in turn causes the level of cellular NAD^ + ^to rise and activate SIRT1 (14). P-AMPK inhibits the activity of genes such as fatty acid synthase (FAS) and sterol regulatory element-binding proteins (SREBPs), which play a role in fatty acid synthesis in the liver or adipose tissue (15, 16). Studies have demonstrated that intracellular cyclic adenosine monophosphate (17) plays a crucial role in AMPK activation and the subsequent activation of SIRT1 (18, 19).

Furthermore, investigations have uncovered that inflammatory indicators like interleukin-6 (IL-6) and tumor necrosis factor-alpha (TNF-α) significantly impact the initiation and progression of NAFLD (20, 21). It has also been shown that the loss of SIRT1 in hepatocytes causes a reduction in the oxidation of fatty acids, the onset of hepatic steatosis, and inflammation (22, 23). Considering the roles of AMPK phosphorylation, SIRT1, autophagy, cAMP, and inflammation in the emergence of hepatic steatosis, they might represent prospective potential targets for treating NAFLD.

## 2. Objectives

This study explored the potential of combining Que and metformin in a HepG2 cell model of hepatic steatosis for the first time. Furthermore, we focused on cAMP/AMPK/SIRT1-mediated autophagy, a cellular process critical to liver health and disease. By examining the effects of Que and metformin on autophagy, we aim to identify new targets for the treatment of NAFLD and contribute to our understanding of the mechanisms underlying the therapeutic effects of these compounds. The findings of this study demonstrated, for the first time, that the combination of Que and metformin relieved hepatic steatosis by stimulating autophagy through the cAMP/AMPK/SIRT1 pathway. This effect was more notable than either treatment alone.

## 3. Methods

### 3.1. Chemicals

Chemicals from Sigma-Aldrich (Germany) were used in the experiments, including Que, metformin, PA (P0500), compound C (CC, P5499), rapamycin (Rapa), DMSO (D2650), bafilomycin A1 (BafA1), forskolin (S1612), KH7, Oil Red O (O0625), and other materials. Antibodies against microtubule-associated protein light chain 3 (LC3) (L7543), AMPK, p62, B-actin, and phosphorylated AMPK (p-AMPK; sc-33524) were supplied by Santa Cruz Biotechnology.

### 3.2. Cell Culture

HepG2 human hepatoma cells used in this study were acquired from Iran’s Pasteur Institute and cultured in DMEM media supplemented with penicillin (100 U/ml), streptomycin (100 μg/ml), and 10% fetal bovine serum (FBS) in a humidified environment at 37°C with 5% CO_2_.

### 3.3. Lipid Content Analysis

HepG2 cells were seeded in six-well plates at a density of 1.2 × 10^6^ cells per well. Oil red O staining was used to determine the total lipid content of cells in order to validate the liver steatosis model. The HepG2 cells were fixed with a 4% paraformaldehyde solution at room temperature for 30 min. The cells were rinsed with distilled water and treated with 60% isopropanol for 10 minutes, then exposed to a new mixture of oil red O working solution made by mixing 300 mg of oil red O powder with 100 ml of 99% isopropanol for 20 minutes. Subsequently, the cells were washed with distilled water and observed under an Olympus upright microscope equipped with a camera (24).

### 3.4. Triglyceride Content Assessment

After being exposed to 0.2 mM P.A., the HepG2 cells were treated for an additional 24 hours with various dosages of Que (0.5 - 10 μM), metformin (0.125 - 5 mM), and the combination of Que (1 and 5 μM) and metformin (0.25 mM) in the presence of BafA1. In RIPA buffer (50 mM Tris-HCl, pH 7.4, 1% Triton X-100, 0.2% sodium deoxycholate, 0.2% SDS, 1 mM Na-EDTA, and 1 mM PMSF), the cells were lysed for 30 minutes after two PBS rinses. In accordance with the protocol, the Biovision triglyceride quantification colorimetric/fluorimetric kit (Biovision Inc., U.S.) was used to measure the amount of intracellular triglyceride.

### 3.5. Cell Viability Assay

The cytotoxicity of metformin, PA, and Que was assessed using the 3-(4,5-dimethylthiazol-2-yl)-2,5-diphenyl-2H-tetrazolium bromide (MTT) assay in order to establish non-toxic levels of the compounds for usage in upcoming experiments. HepG2 cells were seeded in 96-well plates at a density of 10^4^ cells per well, incubated overnight to adhere the plates, and then treated with various concentrations of metformin (0.125 - 5 mM), Que (0.5 - 150 μM), and PA (0.12 - 0.5 mM) for 24 hours. The ELISA reader was used to determine the absorbance at a wavelength of 570 nm after adding MTT solution, incubating for four hours, and dissolving formazan crystals in DMSO.

### 3.6. Real-time PCR

Real-time PCR was used to analyze the expression of the Beclin-1, FAS, SREBP-1c, and LC3 genes. Que (1 and 5 μM) was administered alone and with metformin (0.25 mM) to PA-induced HepG2 cells. RNA was extracted using the RNeasy mini kit (Qiagen, Germany), and cDNA was produced by reverse transcription of the RNA (Bio FACT, Daejeon, South Korea). The SYBR Green Master Mix kit (Ampliqon, Denmark) and the specific primers (Table 1) performed real-time PCR. The 2^-∆∆Ct^ method was utilized to evaluate the outcomes, with the GAPDH gene as the reference gene.

**Table 1.** Real-time PCR Primer Sequences

Gene Name	Forward Primer	Reverse Primer
**LC3**	5’-AAGGCGCTTACAGCTCAATG-3’	5’-CTGGGAGGCATAGACCATGT-3’ (25)
**Beclin-1**	5’-AGCTGCCGTTATACTGTTCTG-3’	5’-ACTGCCTCCTGTGTCTTCAATCTT-3’ (26)
**FAS**	5’- GTGAGGCTGAGGCTGAGAC-3’	5’- GGCACGCAGCTTGTAGTAGA-3’ (27)
**SREBP-1c**	5’-CCATGGATTGCACTTTCGAA-3’	5’- GGCCAGGGAAGTCACTGTCTT-3’ (28)
**GAPDH**	5’-CAAATTCCATGGCACCGTCAAG-3’	5’-AGAGATGATGACCCTTTTGGCT-3’ (29)

### 3.7. Measuring LDH Release

In HepG2 cells, cell death was induced with PA 0.5 mM. Following that, the LDH release assay was employed to assess the effects of metformin (0.25 mM), Que (1 and 5 μM), and their combination on PA-stimulated cell death in the presence of Rapa (100 nM) and sirtinol (100 μM). One hour was spent treating cells with various doses of substances after exposure to 0.5 mM PA overnight in 24-well plates of cells at a density of 1 × 10^5^. The culture medium was then taken out, and LDH was measured using an LDH assay kit (Thermo Scientific Inc., NC9674653) per the manufacturer’s instructions. Finally, the absorbance at 510 nm was measured with an ELISA plate reader.

### 3.8. Measurement of Inflammatory Cytokines

Palmitic acid (0.2 mM), Que (1 and 5 μM), and the combination of Que and metformin (0.25 mM) were administered to the HepG2 cells. The IL-1β, TNF-α, and IL-6 cytokines level was measured in HepG2 cell supernatant (from 1 × 10^5^/24-well plates) using ELISA kits (Sigma-Aldrich, Germany) according to the protocol. Finally, a standard curve was used to determine the precise cytokine levels.

### 3.9. Western Blotting Analysis

HepG2 cells were seeded in six-well culture plates and left to adhere to the plates overnight. Then, the following agents were used to treat the cells: CC, an autophagy inducer; PA; Que; Que + metformin; Sirtinol, a SIRT1 inhibitor. Centrifuging the cells at 412 g for 10 min allowed the cells to be collected and prepared as whole-cell lysates. The supernatant from centrifugally separated lysates was used for western blot analysis after the cells were suspended in RIPA lysis buffer (Thomas Scientific Inc., USA). The samples were electroblotted onto a polyvinylidene fluoride (PVDF) membrane (Millipore, United States) after being loaded into a 10% SDS-PAGE with 40 g of total protein per lane. After being blocked in 5% nonfat milk, the membranes were subjected to specific antibodies against p62, B-actin, LC3-I, LC3-II, AMPK, and p-AMPK. Subsequently, secondary antibodies were incubated with the membranes. Immunoblots were detected using a chemiluminescent kit (SuperSignal, Thermo Fisher Scientific, UK).

### 3.10. cAMP Level Assessment

The total level of cAMP molecules in HepG2 cells was determined using an enzyme immunoassay kit (Stratagene, La Jolla, California). The PA-induced cells, in the presence of forskolin (an AC activator) and KH7 (an AC inhibitor), received metformin (0.25 mM) and Que (1 μM). Then the cell pellets were then suspended in the lysis buffer. The amount of cAMP in HepG2 cells was quantified according to the instructions provided with the kit. In this technique, the cAMP-specific antibody has a limited number of binding sites, and unlabeled cAMP competes with peroxidase-labeled cAMP for binding to the antibody.

### 3.11. Statistical Analyses

Quantitative data are shown using the mean ± SD of three experiments. A post hoc analysis was carried out after using one-way ANOVA to compare the variations between the treatments. Statistical software from SPSS, version 13.0, was used for the analysis. P < 0.05 was used to define a statistically significant difference between treatments.

## 4. Results

### 4.1. Que and Metformin Combination Decreasing the Amount of TG in the HepG2 Cells’ Hepatic Steatosis Model

**Figure 1.**
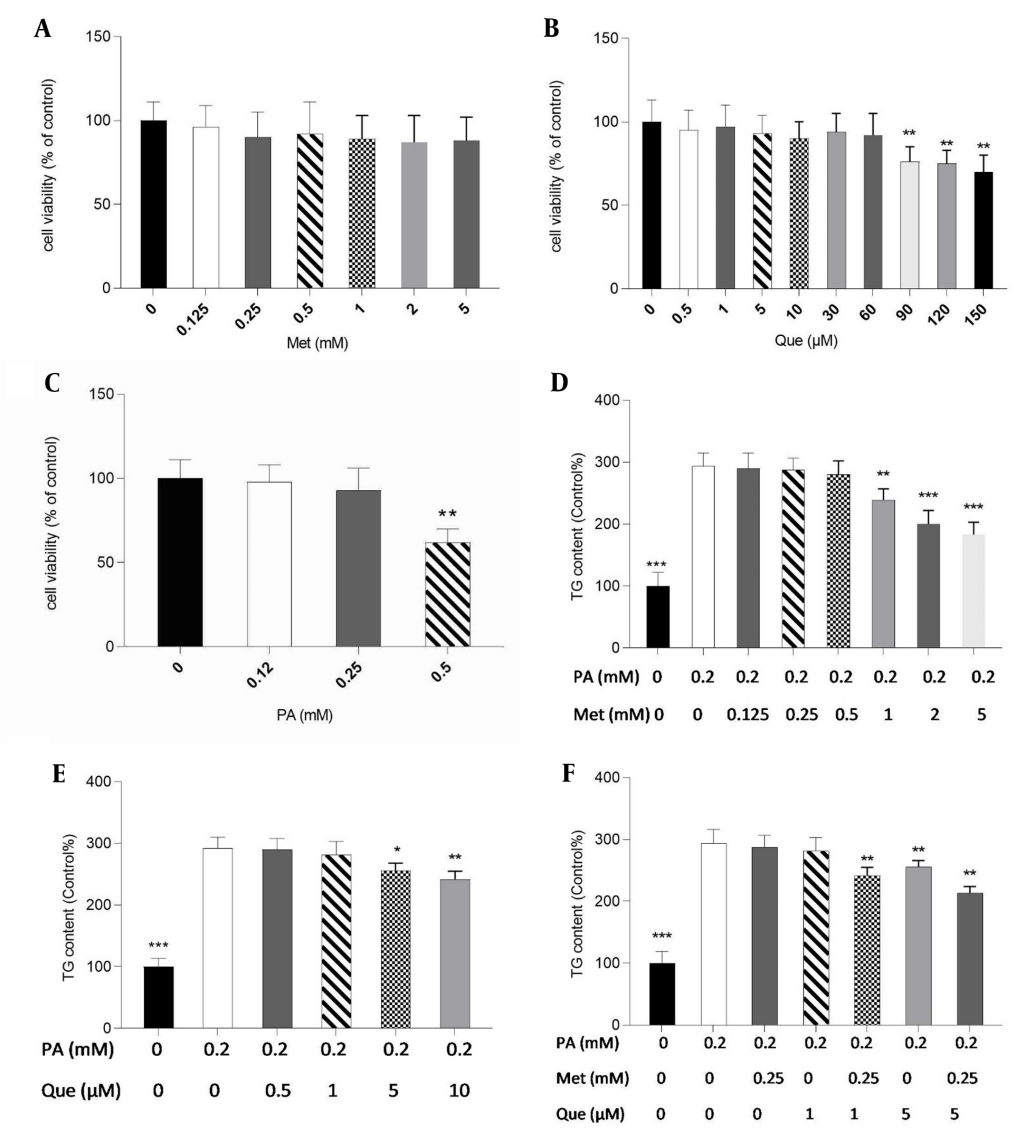
Quercetin (Que) and metformin (Met) combination lowered triglyceride (TG) level in the hepG2 cell model of hepatic steatosis. First, the 3-(4,5-dimethylthiazol-2-yl)-2,5-diphenyl-2H-tetrazolium bromide (MTT) assay was used to assess the cytotoxicity of A, Met; B, Que; and C, palmitic acid (PA). Non-toxic concentrations of the compounds were used for subsequent tests. The intracellular TG level was then determined in response to D, Met; E, Que; and F, met- Que after cells were exposed to PA at 0.2 mM for 24 hours. Data are displayed as mean ± SD (n = 3). * P < 0.05, ** P < 0.01, *** P < 0.001 compared to PA-induced cells. The one-way ANOVA and Tukey’s post hoc test (P < 0.05) show a significant statistical difference between the data and controls.

Initially, for subsequent tests, non-toxic doses of metformin, Que, and PA were determined in HepG2 cells using the MTT assay. Metformin at concentrations up to 5 mM, Que (60 μM), and PA (0.25 mM) did not significantly affect cell viability, as shown in Figure 1A - C. Therefore, 0.2 mM of PA was selected to create a hepatic, non-toxic steatosis model in HepG2 cells. Metformin significantly reduced TG level in PA-induced cells at 1 to 5 mM (Figure 1D). Additionally, the results showed that the TG content was dramatically lessened in the treatment with Que alone at doses of 5 and 10 μM (Figure 1E). As shown in Figure 1F, metformin at 0.25 mM and Que at 1 μM alone was not able to significantly change the TG level; however, in the combination treatment, they significantly diminished TG content compared to the control cells. Furthermore, Que at 5 mM in combination with metformin 0.25 mM reduced TG levels significantly (Figure 1F).

### 4.2. Induction of SIRT1-dependent Autophagy by Que-metformin in PA-induced HepG2 Cells Alleviating Hepatic Steatosis

**Figure 2.**
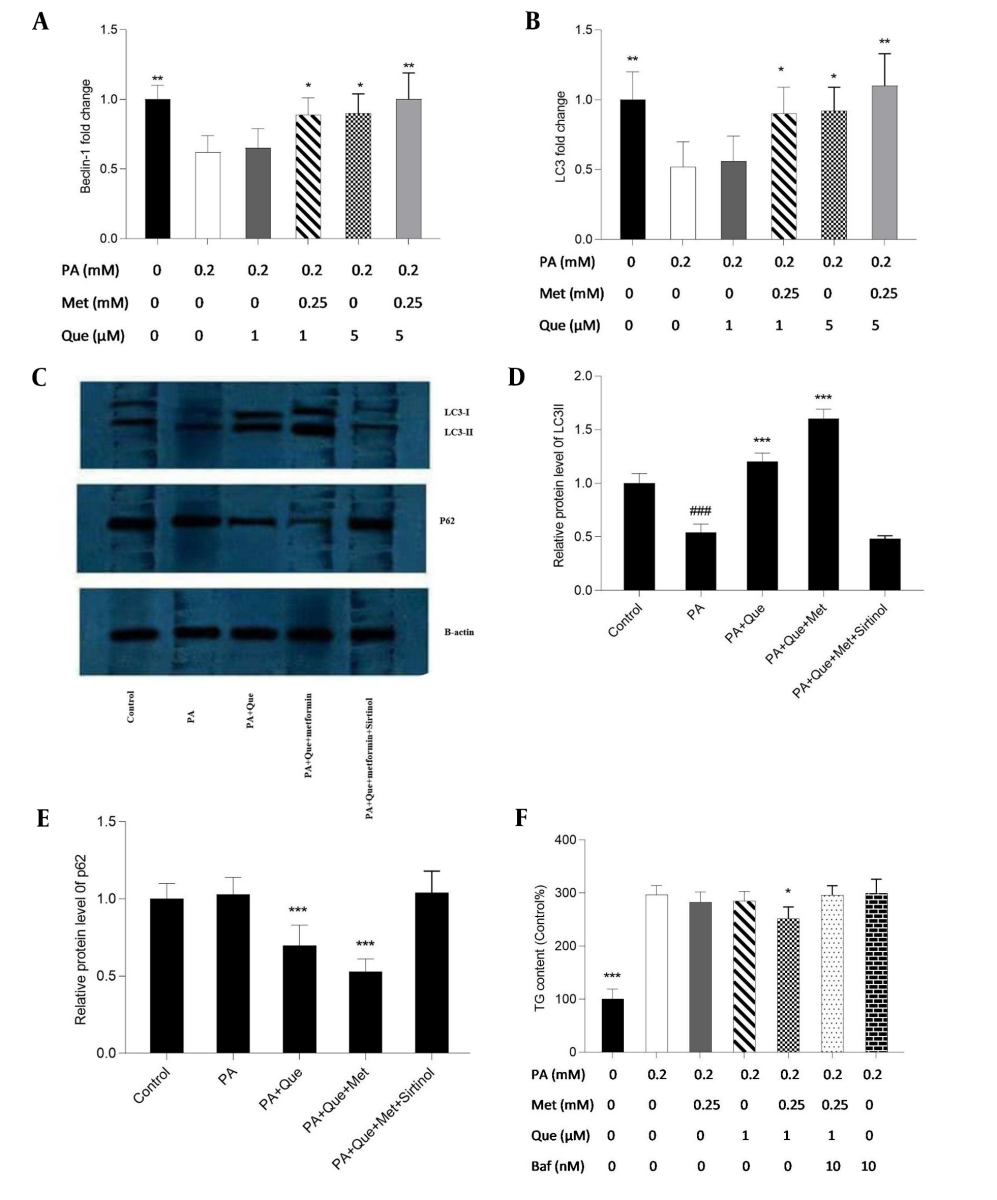
Quercetin (Que)-metformin caused Sirtuin 1 (SIRT1)-dependent autophagy in palmitic acid (PA)-induced HepG2 cells. Real-time PCR was used to measure A, the Beclin-1; B, LC3 genes’ expression in response to Que (1 and 5 μM) and its combination with metformin (0.25 mM). Cells were treated with metformin and Que for an additional 2 hours after exposure to PA (0.2 mM) for 24 hours. After cDNA synthesis and RNA extraction, real-time PCR was performed. Western blot analysis was performed to investigate the impact of different treatments on the expression of LC3-I, II, and p62 proteins. The effects of PA (0.2 mM), PA + Que (5 μM), and PA + Que + metformin (0.25 mM) were examined in the absence and presence of sirtinol, an inhibitor of SIRT1. C, The results indicated that this combination’s induction of autophagy relies on the activity of SIRT1. Quantitative analysis of western blotting data was performed using ImageJ software. # P < 0.05 compared with untreated unstimulated control cells; D and E, * P < 0.05, ** P < 0.01, *** P < 0.001 compared with PA-stimulated untreated cells. D, To explore the role of autophagy in the lipid-lowering effects of Que-metformin, the impact of the autophagy inhibitor Baf was examined, which demonstrated that in the presence of Baf, TG levels did not significantly decrease, suggesting that autophagy plays a role in the ability of Que-metformin to lower lipid levels. * P < 0.05, ** P < 0.01, *** P < 0.001 as compared to cells induced by PA. The data are presented as the mean ± standard deviation (n = 3). The statistical analysis was performed using a one-way ANOVA followed by Tukey’s post hoc test (P < 0.05).

The expression of the Beclin-1 and LC3 genes was examined using real-time PCR to evaluate the impacts of Que-metformin on the autophagy induction in the hepatic steatosis model of HepG2 cells. We also used western blotting analysis to assess the expression of the LC3-I and II proteins and p62, a traditional autophagy receptor (30). The LC3-II protein is formed by LC3-I during autophagy. When lysosomes and autophagosomes fuse, LC3-II is then translocated to the autophagosome membrane and destroyed together with other components of autophagosomes (31). As shown in Figure 2A and B, Que at a concentration of 1 μM did not affect the Beclin-1 and LC3 gene’s expression compared to PA-induced cells, but Que combined with metformin (0.25 mM) significantly increased the expression of both genes. In addition, Que at a dose of 5 μM alone significantly enhanced the expression of Beclin-1 and LC3 genes, which were more significant in combination with metformin (Figure 2A and B). Western blot analysis showed that the presence of Que increased the expression of LC3 I and II and the degradation of p62. This effect was more pronounced when Que was combined with a concentration of 0.25 mM metformin (Figure 2C). Sirtinol, a SIRT1 inhibitor, was used at a concentration of 10 μM in a western blotting assay to determine if SIRT1 is necessary for the induction of autophagy by Que-metformin. According to Figure 2C, adding sirtinol at a concentration of 10 μM counteracted the autophagy-promoting effects of Que-metformin and suppressed the expression of LC3 I and II, as well as the degradation of p62.

**Figure 3.**
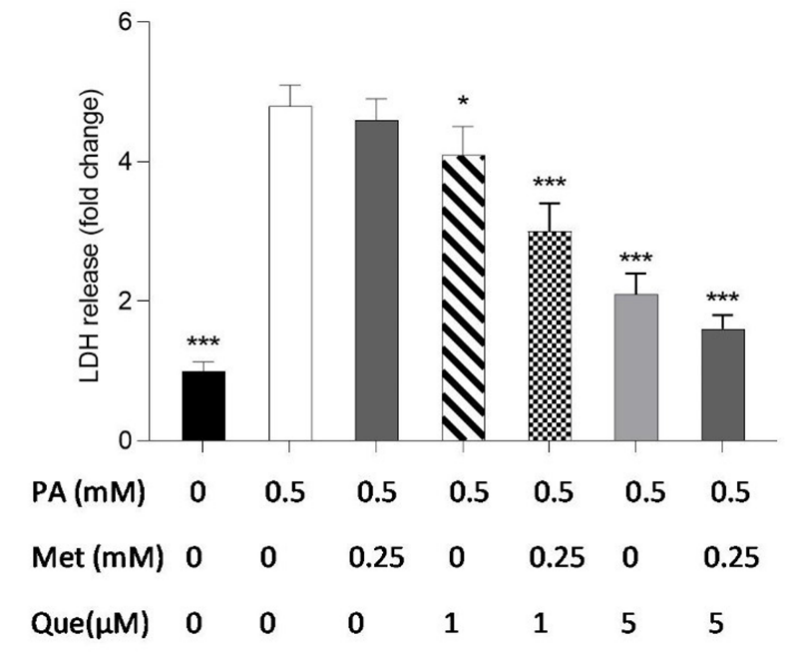
The combination of quercetin (Que) and metformin protected HepG2 cells against palmitic acid (PA)-induced cell death. Palmitic acid at a concentration of 0.5 mM was used to induce cell death. Subsequently, the protective effects of Que (1 and 5 μM), metformin (0.25 mM), and the combination of Que-metformin against PA-induced cell death were investigated. LDH release assay was employed to measure cell death. The results are presented as the mean ± standard deviation (n = 3), and statistical significance was indicated by * P < 0.05, ** P < 0.01, and *** P < 0.001 compared to cells induced by PA. The statistical analysis, conducted using the one-way ANOVA and Tukey’s post hoc test (with a significance level of P < 0.05), indicates a significant difference between the data and control groups.

Furthermore, to investigate if the autophagy induced by Que-metformin plays a role in improving hepatosteatosis, Baf was employed to inhibit autophagy and assess its influence on reducing hepatic steatosis. Baf was utilized to reduce autophagy and evaluate its impact on reducing hepatic steatosis as an autophagy inhibitor. Que (1 μM) and metformin (0.25 mM), as shown in Figure 3D, significantly decreased TG levels compared to the control, whereas the TG-lowering effects of Que-metformin were blocked in the presence of Baf (10 nM).

### 4.3. HepG2 Cells Protected from PA-induced Cell Death by Que-Metformin

The LDH release kit was used to test how well metformin, Que, and their combination protected HepG2 cells against PA-induced cell death. As shown in Figure 3, monotherapy with metformin (0.25 mM) in PA-induced cells did not significantly reduce LDH release or cell death compared to the control cells, but combined with Que (1 and 5 μM) significantly suppressed LDH release. The outcomes also demonstrated that LDH release in PA-induced cells was significantly diminished by Que (5 μM) individually and combined with metformin (0.25 mM).

### 4.4. HepG2 Cells Protected Against PA-induced Cell Death by Que-Metformin’s Autophagy Induction

**Figure 4.**
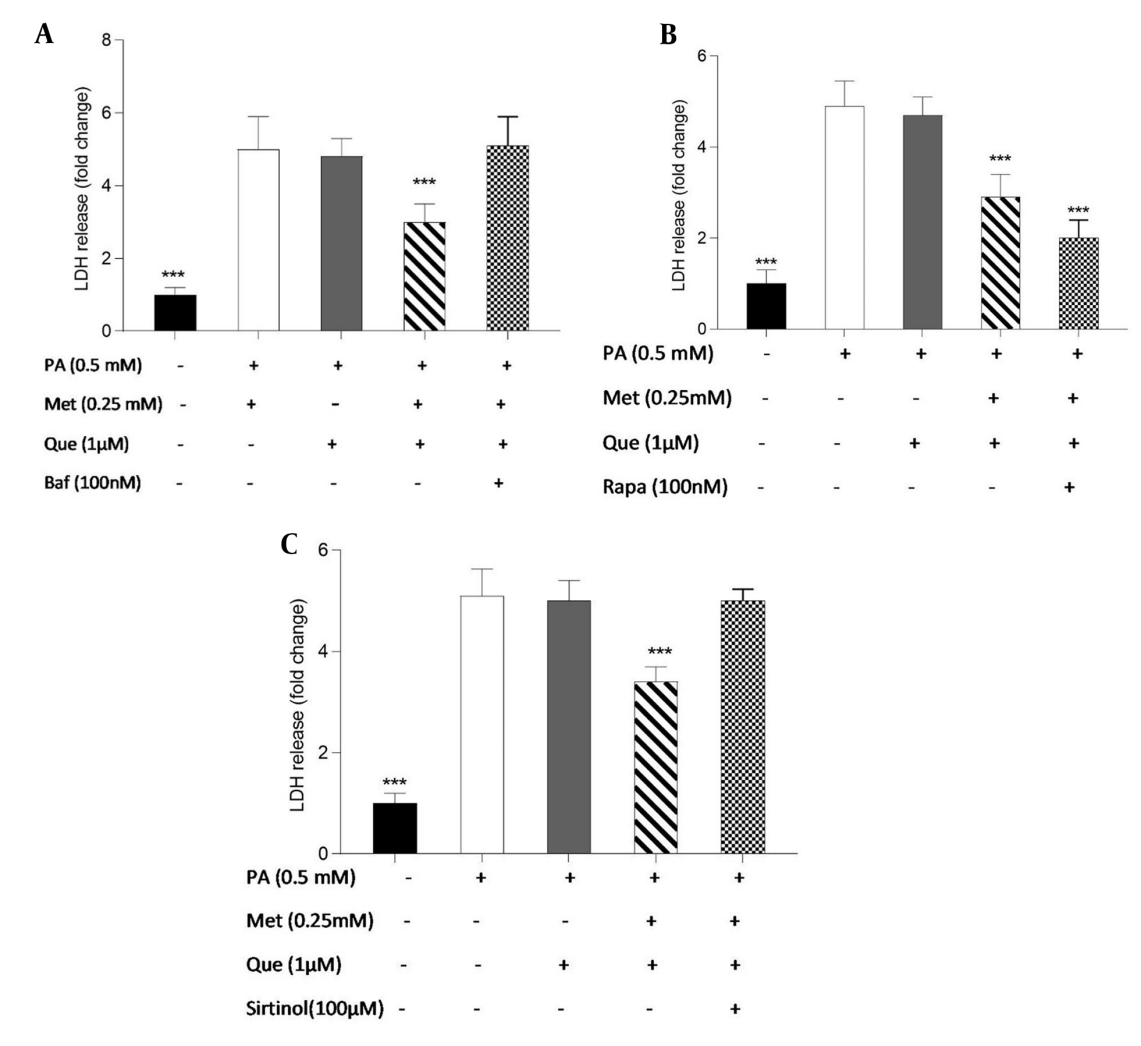
Quercetin (Que)-metformin-induced autophagy in hepatocytes protected cells from palmitic acid (PA)-induced cell death. The protective effects of autophagy induction by Que-metformin against PA-induced cell death were assessed using the LDH release assay in the presence of A, bafilomycin (Baf); B, Rapa; and C, sirtinol. After 16 hours, the LDH level was assessed. The means ± SD of three or more independent batches of cells is used to display all values. * P < 0.05, ** P < 0.01, and *** P < 0.001 were significant compared to the PA-induced cells. Values are displayed as mean ± SD (n = 3). There is a statistically significant difference between the data and controls, as shown by the one-way ANOVA and Tukey’s post hoc test (P 0.05). Que and metformin reduced the level of inflammatory cytokines in a hepatic steatosis model.

The involvement of autophagy in the protective effects of Que-metformin against PA-induced cell death was assessed using the LDH release test. Following exposure to PA (0.5 mM), we conducted cell treatment experiments with and without the addition of Baf (100 nM), Rapa (100 nM), and sirtinol (100 μM). After 16 hours, LDH release was measured. Baf inhibits autophagic vesicle formation by inhibiting the fusion of lysosomes and autolysosomes (32). Rapa can effectively trigger autophagy by inhibiting mTOR activity (33). As shown in Figure 4A, Que (5 M) could not stop the cell death and LDH release caused by PA. However, compared to cells that had been stimulated by PA, LDH release was significantly reduced when Que and metformin (0.25 mM) were combined. The LDH level was not significantly changed in the presence of Baf, an autophagy inhibitor. Que at 1 μM did not significantly reduce LDH release, whereas in combination with metformin (0.25 mM) was able to suppress LDH release and cell death considerably. Que-metformin dramatically reduces the LDH release when Rapa, an autophagy inducer, is present compared to control cells (Figure 4B). According to Figure 4C, the combination of Que (1 μM) and metformin (0.25 mM) significantly reduced LDH release and cell death compared to the control; however, this combination had no effect on the LDH level when sirtinol, a SIRT1 inhibitor, was present, confirming once more that Que-metformin’s autophagy induction is SIRT1-dependent.

**Figure 5.**
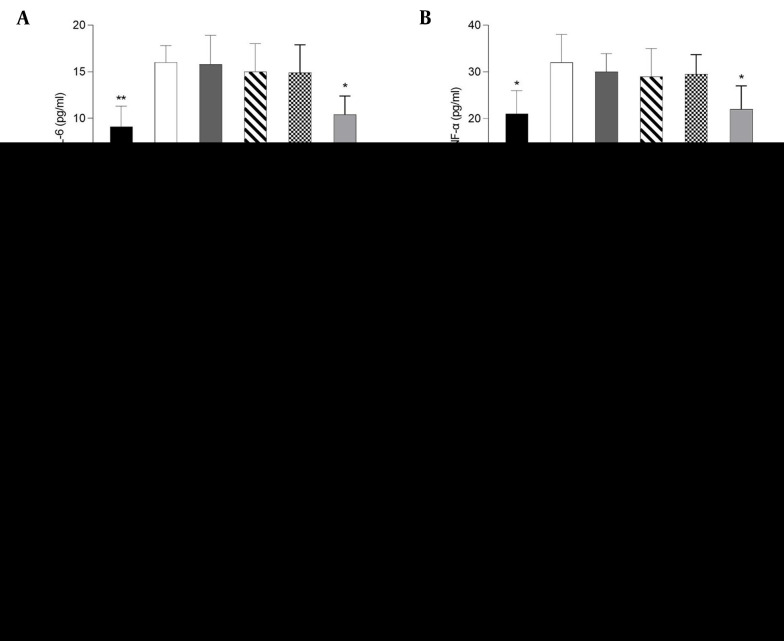
Quercetin (Que)-metformin reducing pro-inflammatory cytokines in HepG2 cells. Using ELISA tests, the impacts of Que-metformin on the inflammatory cytokines in the hepatic steatosis model were evaluated. For 24 hours, the cells were subjected to an exposure of 0.25 mM palmitic acid and subsequently treated with Que (1 and μM) in isolation and in combination with metformin (0.25 mM) for 6 hours. The levels of A, IL-6; B, TNF-α; and C, IL-1B in the cell supernatants were assessed. * P < 0.05, ** P < 0.01, and *** P < 0.001 when compared to the palmitic acid (PA)-treated cells. The data is represented as the mean ± SD (n = 3). The results of the one-way ANOVA and Tukey’s post hoc test (P < 0.05) indicate a statistically significant difference between the data and controls. Que-metformin downregulated sterol regulatory element-binding protein (SREBP)-1c and fatty acid synthase (FAS) gene expression in PA-induced HepG2 cells.

ELISA tests were also used to examine the effects of Que-metformin on the inflammatory response brought on by PA Figure 5A - C shows that only metformin (0.25 mM) and Que (5 μM) were able to decrease the level of pro-inflammatory cytokines significantly.

**Figure 6.**
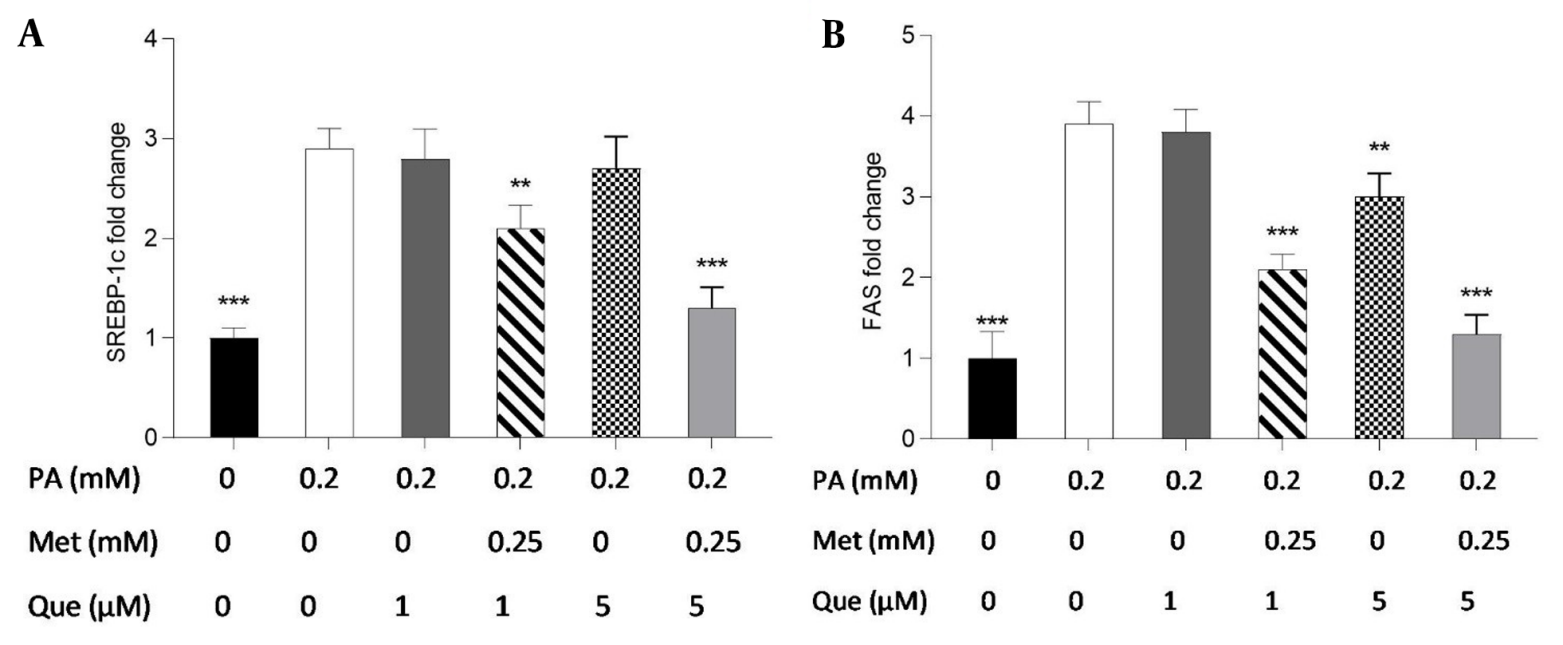
The administration of quercetin (Que)-metformin resulted in a reduction of lipogenic gene expression in HepG2 cells induced with palmitic acid (PA). Real-time PCR was performed to investigate the effects of PA (0.2 mM), metformin (0.25 mM), and Que (1 and μM) on A, sterol regulatory element-binding protein (SREBP)-1c; and B, fatty acid synthase (FAS). After cell treatment with Que and metformin, the expression of both genes was reduced in PA-induced HepG2 cells. With three independent experiments, the data represent means ± SD compared to the PA-induced cells, * P < 0.05, ** P < 0.01, *** P < 0.001. Values are shown as the mean ± SD (n = 3). The one-way ANOVA and Tukey’s post hoc test (P < 0.05) show a significant statistical difference between the data and controls. Que-metformin raised p-AMPK and cAMP levels in PA-induced HepG2 cells.

The present study employed real-time PCR to investigate the expression of the FAS and SREBP-1c genes and to ascertain the molecular underpinnings of the TG-lowering effects of Que-metformin. According to the findings, SREBP-1c gene expression was unaffected by Que (1 and 5M) alone. However, when Que was combined with metformin, it suppressed the expression of the SREBP-1c gene compared to cells induced by PA (Figure 6A). As shown in Figure 6B, Que (1 μM) had no effect on the expression of the FAS gene. However, when combined with metformin (0.25 mM), it could reduce the expression of this gene. Moreover, Que at 5 μM downregulated the expression of the FAS gene, which was more significant in combination with metformin (Figure 6B).

**Figure 7.**
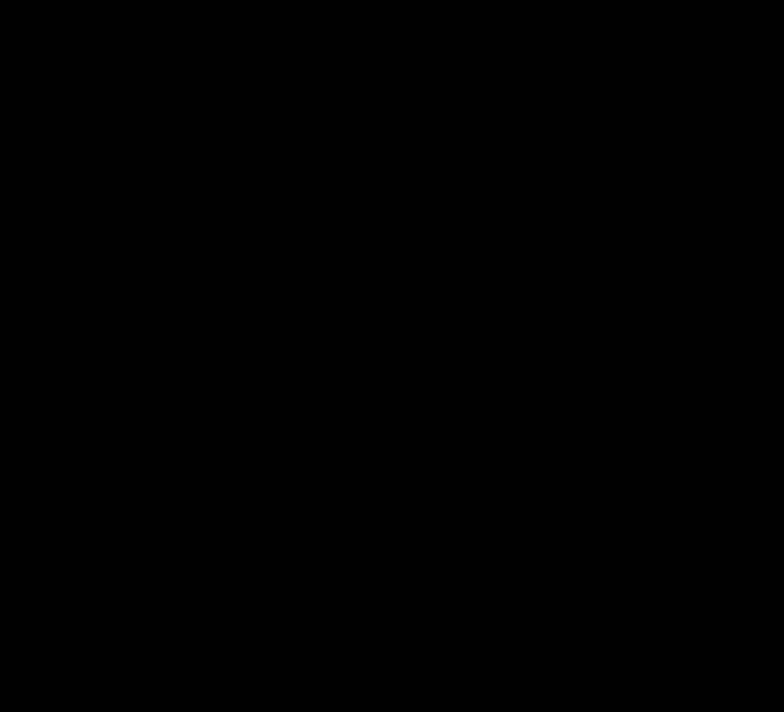
cAMP and p-AMP-activated protein kinase (AMPK) levels were increased by quercetin (Que)-metformin in PA-induced HepG2 cells. A, The impact of Que-metformin on the levels of AMPK and p-AMPK was assessed in the hepatic steatosis model of HepG2 cells utilizing the western blotting technique. The cells were subjected to various treatments, including PA (0.25 mM), PA + Que (5 μM), and PA + Que + metformin (0.25 mM), while the compound C (CC) compound was incorporated as a negative control. Quantitative analysis of western blotting data was performed using ImageJ software. # P < 0.05 compared with untreated unstimulated control cells; B and C, * P < 0.05, ** P < 0.01, *** P < 0.001 compared with PA-stimulated untreated cells. To quantify cAMP levels, cells were subjected to PA (0.2 mM) stimulation for 24 hours, followed by exposure to metformin (0.25 mM) and Que (1 μM) in combination with individual drugs, with or without KH7 (adenylyl cyclase inhibitor, 10 μM) and forskolin (adenylyl cyclase activator, 12 μM) as negative and positive controls, respectively. The cells were then lysed, and 100 μL aliquots of purified lysate were utilized for the cAMP test. The obtained data were presented as mean ± SD (n = 3). Statistical analyses revealed that * P < 0.05, ** P < 0.01, *** P < 0.001 compared to PA-induced cells. The one-way ANOVA and Tukey’s post hoc test (P < 0.05) demonstrated a significant statistical difference between the data and controls.

Que’s impact on AMPK and p-AMPK, alone and in combination with metformin, was evaluated using Western blotting analysis. As illustrated in Figure 7A, Que (5 μM) exhibited an augmentation in the levels of p-AMPK compared to PA-induced cells, and this effect was more pronounced in conjunction with metformin (0.25 mM). However, the total level of AMPK was not modified by the compounds. Additionally, the negative control, AMPK inhibitor CC (10 μM), repressed the expression of p-AMPK. The content of cAMP in response to the compounds was also evaluated. As depicted in Figure 7B, Que (1 μM) had no discernible effect on cAMP content, but combined with metformin (0.25 mM), it elevated the cAMP level. The adenylyl cyclase inhibitor KH7 (10 μM) was utilized to assess the cAMP-dependent action of Que-metformin. Furthermore, the activator of adenylyl cyclase, forskolin (10 μM), was employed to serve as a positive control. In the presence of KH7, Que-metformin did not significantly elevate the levels of cAMP compared to PA-induced cells.

## 5. Discussion

Non-alcoholic fatty liver disease is the most prevalent liver disease, ranging from hepatic steatosis to NASH, which is associated with lipotoxicity in the liver and still has no approved drug for treatment (34, 35). Novel medications and therapeutic strategies are therefore required to treat NAFLD.

Recent studies have revealed that dysregulation of autophagy contributes to the development of NAFLD; as a result, it may have a potential therapeutic target for the treatment of NAFLD (36-38). In the regulation of hepatic lipid metabolism via autophagy, SIRT1/AMPK plays a crucial function (39). Several studies have shown that Que (40, 41) and metformin (42, 43) have promising lipid-lowering effects by modulating NAFLD autophagy. However, the beneficial effects of the combination of Que and metformin on NAFLD have yet to be investigated. In the current study, the combination of Que and metformin was utilized for the first time to assess their effects on lipid accumulation in the hepatic steatosis model of HepG2 cells as well as to clarify the underlying mechanism of action.

In this research, PA was utilized to create a model of hepatic steatosis in HepG2 cells. The results indicated a significant decrease in TG content in PA-induced HepG2 cells when treated with metformin at concentrations above 1 mM and Que at 5 and 10 μM doses. Metformin (0.25 mM) and Que (1 μM) in combination treatment, which were ineffective when used alone, could significantly reduce TG buildup. In conclusion, the combination of Que and metformin showed more remarkable lipid-lowering effects than monotherapy. These compounds could be used at lower doses with fewer side effects and greater efficacy. In line with these results, previous studies have indicated that metformin (44) and Que (45) can reduce TG in HepG2 cells. To further examine the mechanism of the lipid-lowering action of Que-metformin on HepG2 cells, the expression of FAS and SREBP-1c lipogenic genes and the involvement of autophagy were determined using real-time PCR and western blot analysis. The results of real-time PCR demonstrated that Que-metformin diminished the expression of lipogenic genes, thereby preventing hepatic TG synthesis. Consistent with these findings, studies have shown that metformin (44, 46) and Que (45) inhibit FAS and SREBP-1c gene expression.

Additionally, Que-metformin remarkably increased the expression of Beclin-1, a key autophagy inducer (47), and LC3, protein-related autophagy (31) in PA-induced HepG2 cells, which was not observed in monotherapy. The western blotting analysis also showed that Que-metformin increased the level of LC3-I, II, and p62 degradation. In this study, the role of SIRT1, an autophagy regulator (48), in autophagy induction by Que-metformin was also evaluated. For this purpose, a selective SIRT1 inhibitor known as sirtinol was employed as a negative control in the western blotting analysis. The results indicated that the effects of the combination on autophagy markers, namely P62 degradation and LC3-I and II elevation, were suppressed in the presence of sirtinol. These findings suggest that Que-metformin induces autophagy in a SIRT1-dependent manner. In a separate experiment, Baf, an autophagy inhibitor, was utilized in the TG-content measurement experiment. The results revealed that Que-metformin could not lower TG content in the presence of Baf. As a result, it can be concluded that Que-metformin improves hepatic steatosis via SIRT1-dependent autophagy induction in HepG2 cells.

Autophagy has been suggested to play a role in the cytotoxicity induced by PA in hepatocytes. One investigation found that PA triggers autophagy responses in hepatic cells via a mechanism involving the activation of JNK2, which antagonizes PA-JNK1-induced cytotoxic effects (49). Another study suggested that autophagy may be a structure-dependent defense model in the early stage of PA intoxication (50). The present study investigated the role of autophagy in the protective effects of Que-metformin against cell death induced by PA. This was accomplished by conducting LDH release assays in the presence of Baf, Rapa, and sirtinol. The results revealed that Que-metformin did not significantly alter LDH levels in the presence of Baf. However, when combined with Rapa, an autophagy inducer, Que-metformin significantly decreased LDH release and cell death. Conversely, this combination failed to lower LDH levels in the presence of sirtinol, indicating that SIRT1 is essential for Que-metformin-induced autophagy. These findings suggest, for the first time, that Que-metformin’s protective effects against PA-induced cell death are mediated by autophagy induction in HepG2 cells. The inhibition of lipogenesis-related genes, specifically FAS and SREBP-1c, which are crucial components in the pathogenesis of NAFLD, has been demonstrated by P-AMPK (51). The expression of p-AMPK was notably increased by Que-metformin in PA-induced hepatocytes, as observed in western blotting analysis, without any alteration in total AMPK. The combination treatment exhibited a more substantial p-AMPK level than the Que treatment alone. The involvement of cAMP in Que-metformin-induced autophagy in HepG2 cells was also investigated, and the results showed that Que-metformin significantly elevated the cAMP level in PA-induced hepatocytes for the first time. To explore the mechanism behind the cAMP increase by Que-metformin, an adenylyl cyclase inhibitor, KH7, was utilized. The adenylyl cyclase enzyme’s participation in enhancing cAMP levels by Que-metformin was demonstrated by the lack of significant increase in cAMP levels in response to Que-metformin in the presence of KH7. As demonstrated in previous studies, IL-6 and TNF-α are significant cytokines in the development of hepatic steatosis (52).

Additionally, autophagy has been associated with hepatocyte inflammatory response (53). The results of ELISA tests have shown that Que-metformin significantly reduces the production of IL-6 and TNF-α, as well as IL-1B proinflammatory cytokines, which is a more significant reduction compared to Que treatment alone. Recent investigations have shown that the deletion of SIRT1 in hepatocytes leads to increased local inflammation (22). It is hypothesized that the anti-inflammatory properties of Que-metformin may be mediated through SIRT1-dependent autophagy induction. Further research is necessary to fully comprehend the role SIRT1 plays in Que-metformin’s anti-inflammatory effects on HepG2 cells.

### 5.1. Conclusions

In conclusion, the present investigation has provided initial evidence that Que-metformin, an innovative combined therapeutic approach, can reduce hepatic steatosis in PA-induced HepG2 cells by stimulating autophagy through the cAMP/AMPK/SIRT1 pathway and diminishing inflammatory cytokines. Nevertheless, additional in vivo studies are required to determine the therapeutic potential of the metformin and Que combination for NAFLD.

## Data Availability

The datasets analyzed during this study are not publicly available.

## References

[1] Koo SH (2013). Nonalcoholic fatty liver disease: molecular mechanisms for the hepatic steatosis.. Clin Mol Hepatol..

[2] El-Agroudy NN, Kurzbach A, Rodionov RN, O'Sullivan J, Roden M, Birkenfeld AL (2019). Are Lifestyle Therapies Effective for NAFLD Treatment?. Trends Endocrinol Metab..

[3] Abenavoli L, Larussa T, Corea A, Procopio AC, Boccuto L, Dallio M (2021). Dietary Polyphenols and Non-Alcoholic Fatty Liver Disease.. Nutrients..

[4] Chen L, Liu J, Mei G, Chen H, Peng S, Zhao Y (2021). Quercetin and non-alcoholic fatty liver disease: A review based on experimental data and bioinformatic analysis.. Food Chem Toxicol..

[5] Kendall DL, Amin R, Clayton PE (2014). Metformin in the treatment of obese children and adolescents at risk of type 2 diabetes.. Paediatr Drugs..

[6] Ozturk ZA, Kadayifci A (2014). Insulin sensitizers for the treatment of non-alcoholic fatty liver disease.. World J Hepatol..

[7] Mazza A, Fruci B, Garinis GA, Giuliano S, Malaguarnera R, Belfiore A (2012). The role of metformin in the management of NAFLD.. Exp Diabetes Res..

[8] Garber AJ, Duncan TG, Goodman AM, Mills DJ, Rohlf JL (1997). Efficacy of metformin in type II diabetes: results of a double-blind, placebo-controlled, dose-response trial.. Am J Med..

[9] Okayasu S, Kitaichi K, Hori A, Suwa T, Horikawa Y, Yamamoto M (2012). The evaluation of risk factors associated with adverse drug reactions by metformin in type 2 diabetes mellitus.. Biol Pharm Bull..

[10] Zhou J, Massey S, Story D, Li L (2018). Metformin: An Old Drug with New Applications.. Int J Mol Sci..

[11] Khambu B, Yan S, Huda N, Liu G, Yin XM (2018). Autophagy in non-alcoholic fatty liver disease and alcoholic liver disease.. Liver Res..

[12] Kitada M, Ogura Y, Koya D, Hayat MA (2016). Role of Sirt1 as a Regulator of Autophagy.. Autophagy: Cancer, Other Pathologies, Inflammation, Immunity, Infection, and Aging..

[13] Ruderman NB, Xu XJ, Nelson L, Cacicedo JM, Saha AK, Lan F (2010). AMPK and SIRT1: a long-standing partnership?. Am J Physiol Endocrinol Metab..

[14] Canto C, Gerhart-Hines Z, Feige JN, Lagouge M, Noriega L, Milne JC (2009). AMPK regulates energy expenditure by modulating NAD+ metabolism and SIRT1 activity.. Nature..

[15] Moslehi A, Hamidi-Zad Z (2018). Role of SREBPs in Liver Diseases: A Mini-review.. J Clin Transl Hepatol..

[16] Xiao Q, Zhang S, Yang C, Du R, Zhao J, Li J (2019). Ginsenoside Rg1 Ameliorates Palmitic Acid-Induced Hepatic Steatosis and Inflammation in HepG2 Cells via the AMPK/NF-kappaB Pathway.. Int J Endocrinol..

[17] de Oliveira CP, Stefano JT, de Siqueira ER, Silva LS, de Campos Mazo DF, Lima VM (2008). Combination of N-acetylcysteine and metformin improves histological steatosis and fibrosis in patients with non-alcoholic steatohepatitis.. Hepatol Res..

[18] Park SJ, Ahmad F, Philp A, Baar K, Williams T, Luo H (2012). Resveratrol ameliorates aging-related metabolic phenotypes by inhibiting cAMP phosphodiesterases.. Cell..

[19] Wan D, Zhou Y, Wang K, Hou Y, Hou R, Ye X (2016). Resveratrol provides neuroprotection by inhibiting phosphodiesterases and regulating the cAMP/AMPK/SIRT1 pathway after stroke in rats.. Brain Res Bull..

[20] Arrese M, Cabrera D, Kalergis AM, Feldstein AE (2016). Innate Immunity and Inflammation in NAFLD/NASH.. Dig Dis Sci..

[21] Duarte N, Coelho IC, Patarrao RS, Almeida JI, Penha-Goncalves C, Macedo MP (2015). How Inflammation Impinges on NAFLD: A Role for Kupffer Cells.. Biomed Res Int..

[22] Purushotham A, Schug TT, Xu Q, Surapureddi S, Guo X, Li X (2009). Hepatocyte-specific deletion of SIRT1 alters fatty acid metabolism and results in hepatic steatosis and inflammation.. Cell Metab..

[23] Yin H, Hu M, Liang X, Ajmo JM, Li X, Bataller R (2014). Deletion of SIRT1 from hepatocytes in mice disrupts lipin-1 signaling and aggravates alcoholic fatty liver.. Gastroenterology..

[24] Babaei Khorzoughi R, Namvarjah F, Teimouri M, Hosseini H, Meshkani R (2019). In-vitro Synergistic Effect of Metformin and Berberine on High Glucose-induced Lipogenesis.. Iran J Pharm Res..

[25] Cheng C, Deng X, Xu K (2018). Increased expression of sterol regulatory element binding protein‑2 alleviates autophagic dysfunction in NAFLD.. Int J Mol Med..

[26] Joseph JJ, Leestemaker-Palmer A, Kazemi S, Danelishvili L, Bermudez LE, Kolla J (2023). Mycobacterium avium Infection of Multinucleated Giant Cells Reveals Association of Bacterial Survival to Autophagy and Cholesterol Utilization.. Cell Microbiol..

[27] Raina V, Gupta S, Yadav S, Surolia A (2013). Simvastatin induced neurite outgrowth unveils role of cell surface cholesterol and acetyl CoA carboxylase in SH-SY5Y cells.. PLoS One..

[28] Fernandez-Alvarez A, Alvarez MS, Gonzalez R, Cucarella C, Muntane J, Casado M (2011). Human SREBP1c expression in liver is directly regulated by peroxisome proliferator-activated receptor alpha (PPARalpha).. J Biol Chem..

[29] San TT, Khaenam P, Prachayasittikul V, Sripa B, Kunkeaw N, Chan-On W (2020). Curcumin enhances chemotherapeutic effects and suppresses ANGPTL4 in anoikis-resistant cholangiocarcinoma cells.. Heliyon..

[30] Liu WJ, Ye L, Huang WF, Guo LJ, Xu ZG, Wu HL (2016). p62 links the autophagy pathway and the ubiqutin-proteasome system upon ubiquitinated protein degradation.. Cell Mol Biol Lett..

[31] Tanida I, Ueno T, Kominami E (2008). LC3 and Autophagy.. Methods Mol Biol..

[32] Shacka JJ, Klocke BJ, Roth KA (2006). Autophagy, bafilomycin and cell death: the "a-B-cs" of plecomacrolide-induced neuroprotection.. Autophagy..

[33] Sarkar S, Ravikumar B, Floto RA, Rubinsztein DC (2009). Rapamycin and mTOR-independent autophagy inducers ameliorate toxicity of polyglutamine-expanded huntingtin and related proteinopathies.. Cell Death Differ..

[34] Kim D, Siddique O, Perumpail BJ, Ahmed A, Wong RJ, Gish RG (2019). Clinical Epidemiology of NAFLD.. Clinical Epidemiology of Chronic Liver Diseases..

[35] Munteanu MA, Nagy GA, Mircea PA (2016). Current Management of NAFLD.. Clujul Med..

[36] Czaja MJ (2016). Function of Autophagy in Nonalcoholic Fatty Liver Disease.. Dig Dis Sci..

[37] Zhang Y, Li K, Kong A, Zhou Y, Chen D, Gu J (2021). Dysregulation of autophagy acts as a pathogenic mechanism of non-alcoholic fatty liver disease (NAFLD) induced by common environmental pollutants.. Ecotoxicol Environ Saf..

[38] Lavallard VJ, Gual P (2014). Autophagy and non-alcoholic fatty liver disease.. Biomed Res Int..

[39] Fulco M, Sartorelli V (2008). Comparing and contrasting the roles of AMPK and SIRT1 in metabolic tissues.. Cell Cycle..

[40] Ashrafizadeh M, Ahmadi Z, Farkhondeh T, Samarghandian S (2022). Autophagy as a molecular target of quercetin underlying its protective effects in human diseases.. Arch Physiol Biochem..

[41] Liu L, Gao C, Yao P, Gong Z (2015). Quercetin Alleviates High-Fat Diet-Induced Oxidized Low-Density Lipoprotein Accumulation in the Liver: Implication for Autophagy Regulation.. Biomed Res Int..

[42] Song YM, Lee YH, Kim JW, Ham DS, Kang ES, Cha BS (2015). Metformin alleviates hepatosteatosis by restoring SIRT1-mediated autophagy induction via an AMP-activated protein kinase-independent pathway.. Autophagy..

[43] Li YL, Li XQ, Wang YD, Shen C, Zhao CY (2019). Metformin alleviates inflammatory response in non-alcoholic steatohepatitis by restraining signal transducer and activator of transcription 3-mediated autophagy inhibition in vitro and in vivo.. Biochem Biophys Res Commun..

[44] Zhu X, Yan H, Xia M, Chang X, Xu X, Wang L (2018). Metformin attenuates triglyceride accumulation in HepG2 cells through decreasing stearyl-coenzyme A desaturase 1 expression.. Lipids Health Dis..

[45] Li X, Wang R, Zhou N, Wang X, Liu Q, Bai Y (2013). Quercetin improves insulin resistance and hepatic lipid accumulation in vitro in a NAFLD cell model.. Biomed Rep..

[46] Zare M, Panahi G, Koushki M, Mostafavi-Pour Z, Meshkani R (2022). Metformin reduces lipid accumulation in HepG2 cells via downregulation of miR-33b.. Arch Physiol Biochem..

[47] Kang R, Zeh HJ, Lotze MT, Tang D (2011). The Beclin 1 network regulates autophagy and apoptosis.. Cell Death Differ..

[48] Ou X, Lee MR, Huang X, Messina-Graham S, Broxmeyer HE (2014). SIRT1 positively regulates autophagy and mitochondria function in embryonic stem cells under oxidative stress.. Stem Cells..

[49] Tu QQ, Zheng RY, Li J, Hu L, Chang YX, Li L (2014). Palmitic acid induces autophagy in hepatocytes via JNK2 activation.. Acta Pharmacol Sin..

[50] Liu W, Li X, Zhou B, Fang S, Ho W, Chen H (2017). Differential induction of apoptosis and autophagy by pyrrolizidine alkaloid clivorine in human hepatoma Huh-7.5 cells and its toxic implication.. PLoS One..

[51] Ahmed MH, Byrne CD (2007). Modulation of sterol regulatory element binding proteins (SREBPs) as potential treatments for non-alcoholic fatty liver disease (NAFLD).. Drug Discov Today..

[52] Jarrar MH, Baranova A, Collantes R, Ranard B, Stepanova M, Bennett C (2008). Adipokines and cytokines in non-alcoholic fatty liver disease.. Aliment Pharmacol Ther..

[53] Kouroumalis E, Voumvouraki A, Augoustaki A, Samonakis DN (2021). Autophagy in liver diseases.. World J Hepatol..

